# ChiroVox: a public library of bat calls

**DOI:** 10.7717/peerj.12445

**Published:** 2022-01-13

**Authors:** Tamás Görföl, Joe Chun-Chia Huang, Gábor Csorba, Dorottya Győrössy, Péter Estók, Tigga Kingston, Kriszta Lilla Szabadi, Ellen McArthur, Juliana Senawi, Neil M. Furey, Vuong Tan Tu, Vu Dinh Thong, Faisal Ali Anwarali Khan, Emy Ritta Jinggong, Melissa Donnelly, Jayaraj Vijaya Kumaran, Jian-Nan Liu, Shiang-Fan Chen, Mao-Ning Tuanmu, Ying-Yi Ho, Heng-Chia Chang, Nurul-Ain Elias, Nur-Izzati Abdullah, Lee-Sim Lim, C Daniel Squire, Sándor Zsebők

**Affiliations:** 1 Department of Zoology, Hungarian Natural History Museum, Budapest, Hungary; 2 National Laboratory of Virology, Szentágothai Research Centre, University of Pécs, Pécs, Hungary; 3 Biodiversity Research Center, Academia Sinica, Taipei, Taiwan; 4 Department of Biological Sciences and Biotechnology, Faculty of Science and Technology, Universiti Kebangsaan Malaysia, Bangi, Selangor, Malaysia; 5 Southeast Asian Bat Conservation Research Unit, Lubbock, TX, United States of America; 6 Hungarian University of Agriculture and Life Sciences, Gödöllő, Hungary; 7 Department of Zoology, Eszterházy Károly Catholic University, Eger, Hungary; 8 Department of Biological Sciences, Texas Tech University, Lubbock, TX, United States of America; 9 Faculty of Resource Science and Technology, Universiti Malaysia Sarawak, Kota Samarahan, Sarawak, Malaysia; 10 Harrison Institute, Kent, United Kingdom; 11 Fauna & Flora International, Phnom Penh, Cambodia; 12 Institute of Ecology and Biological Resources, Vietnam Academy of Science and Technology, Hanoi, Vietnam; 13 Graduate University of Science and Technology, Vietnam Academy of Science and Technology, Hanoi, Vietnam; 14 Operation Wallacea Ltd., Lincolnshire, United Kingdom; 15 Proyecto CUBABAT, Matanzas, Cuba; 16 Global Entrepreneurship Research & Innovation Center, Universiti Malaysia Kelantan, Pengkalan Chepa, Kota Bharu, Malaysia; 17 Department of Forestry and Natural Resources, National Chiayi University, Chiayi, Taiwan; 18 Center for General Education, National Taipei University, New Taipei City, Taiwan; 19 Formosan Golden Bat’s Home, Yunlin, Taiwan; 20 School of Biological Sciences, Universiti Sains Malaysia, Penang, Malaysia; 21 School of Distance Education, Universiti Sains Malaysia, Penang, Malaysia; 22 Department of Educational Psychology & Leadership, Texas Tech University, Lubbock, TX, United States of America; 23 Department of Systematic Zoology and Ecology, Eötvös Loránd University, Budapest, Hungary; 24 Institute of Ecology and Botany, Centre for Ecological Research, Vácrátót, Hungary

**Keywords:** Bats, Chiroptera, Database, Call library, Echolocation, Acoustics, Survey, Monitoring

## Abstract

Recordings of bat echolocation and social calls are used for many research purposes from ecological studies to taxonomy. Effective use of these relies on identification of species from the recordings, but comparative recordings or detailed call descriptions to support identification are often lacking for areas with high biodiversity. The ChiroVox website (https://www.chirovox.org) was created to facilitate the sharing of bat sound recordings together with their metadata, including biodiversity data and recording circumstances. To date, more than 30 researchers have contributed over 3,900 recordings of nearly 200 species, making ChiroVox the largest open-access bat call library currently available. Each recording has a unique identifier that can be cited in publications; hence the acoustic analyses are repeatable. Most of the recordings available through the website are from bats whose species identities are confirmed, so they can be used to determine species in recordings where the bats were not captured or could not be identified. We hope that with the help of the bat researcher community, the website will grow rapidly and will serve as a solid source for bat acoustic research and monitoring.

## Introduction

Bats are the second most specious group of mammals with more than 1400 known species (Simmons & Cirranello, 2020). They play crucial roles in ecosystems and provide important ecosystem services to humans through suppression of agricultural pests, seed dispersal and pollination (Kunz et al., 2011). More than one-third of bat species listed by the International Union for Conservation of Nature (IUCN) are considered threatened or data deficient and information on species’ distributions, habitat use, and population trends are required to ensure appropriate conservation measures for these taxa (Frick, Kingston & Flanders, 2020).

Due to their elusive nature, bats are among the least known mammals. This is especially true for tropical bat species. Observation of bats is challenging because they are active at night and roost in crevices or inaccessible places during the day. There are several methods to determine species occurrence and abundance, for example by catching individuals with mist nets, harp traps, or visiting roosts. However, these methods cannot be used equally for various species and usually preclude observation of natural behaviors (*e.g.*, Larsen et al., 2007; MacSwiney, Clarke & Racey, 2008; Kingston, 2013; Marques et al., 2013; Tanshi & Kingston, 2021). Approximately 86% of bat species use echolocation for navigation, and the calls of many species are sufficiently intense that they can be recorded while the bat is flying freely in the natural environment. This provides an opportunity for researchers to “eavesdrop” on bats in their natural habitats, such that acoustic methods have become a mainstay in bat research in recent decades (Zamora-Gutierrez et al., 2021).

Acoustic approaches have several advantages. They can be used without disturbing bats and automatic recorders can be deployed for several days or months. Multiple recording devices can also simultaneously be used across large areas for comparisons of land use (*e.g.*, Frick, 2013; Gibb et al. 2019). However, acoustic methods also have disadvantages, the most critical being that it is generally challenging to identify all calls to species level, especially in areas with high bat diversity. Echolocation calls of many species, including co-distributed taxa, are similar and overlap in acoustic parameters, making it difficult to distinguish among species. This is compounded by within species and even within individual variations in call parameters, as species’ calls vary geographically and bats commonly adjust calls in the course of a call sequence (Russo & Voigt, 2016; Rydell et al., 2017; Russo, Ancillotto & Jones, 2018; Goerlitz, 2018). For accurate identification of species from recordings, there is an urgent need for large databases that provide greater information on within- and between-species variation in echolocation calls, especially in tropical areas.

In the last decade, the number of studies detailing the acoustic parameters of tropical bat calls has increased (*e.g.*, Hughes et al., 2011; Phauk, Sarith & Furey, 2013; Zamora-Gutierrez et al., 2016; Hackett, Holderied & Korine, 2017; Monadjem et al., 2017; Raman & Hughes, 2021; López-Bosch et al., 2021; McArthur & Khan, 2021). These publications primarily focus on describing acoustic parameters that can be helpful for identifying bats in many cases, but definitions and terminology often differ between studies, which hinders comparisons. Moreover, call parameters are commonly reported as mean values and standard deviations/standard errors, which can be difficult to use for species identification. In contrast, access to verified reference recordings for species that can then be compared to assess anonymous calls in the same software environment is much more efficient and reliable.

The need for echolocation call libraries has long been suggested (Karine & Kalko, 2001). A number of bat call collections have been published in the last two decades, but only a few are still maintained and even fewer cover large geographic areas (Table 1).

**Table 1:** A list of bat sound libraries based on Walters et al. (2013) and Zamora-Gutierrez et al. (2021).

Name	No. species	No. records	Country/ Region	Reference	Download
EchoBank	297	3531	Worldwide	Collen (2012)	not online
Bat Conservation Trust Sound Library	15	27	Great Britain	http://www.bats.org.uk	only members
Cornell lab of ornithology –Macaulay library	29+	258+	Worldwide	http://www.macaulaylibrary.org	no
British library –British sound archive	139+	700+	Europe	https://sounds.bl.uk	no
Nepal Bat Call Library	15	15	Nepal	http://smcrf.org/resource/nepalbatcall	no
BioSounds –Sumatran Chiroptera	16	16	Sumatra, Indonesia	https://soundefforts.uni-goettingen.de/biosounds/collection/show/19	no
Bat Calls of New South Wales	31	1200+	New South Wales, Australia	https://www.environment.nsw.gov.au/surveys/Batcalls.htm	yes
Avisoft Bioacoustics	26	62	Europe	http://www.batcalls.com	yes
Morcegoteca	17	27	Brazil	https://ppbio.inpa.gov.br/en/Bat_Library	yes
Sonozotz	69	1960	Mexico	Soon through the CONABIO portal	not yet
ChiroVox	192	3902	Worldwide	http://www.chirovox.org	yes

One of the most significant databases is EchoBank, which contains thousands of bat recordings, although unfortunately the sound files themselves are not available online (Collen, 2012). The most recent project is Sonozotz, which is based on a thorough survey in a megadiverse country, Mexico, and will hopefully be available online soon (Zamora-Gutierrez et al., 2020). Online libraries are currently available only for regional levels and are not continuously updated with new recordings.

Recognizing the need for a large and curated call library, we created the ChiroVox website (https://www.chirovox.org.) whose purpose is to act as a freely available collection of reference bat calls without geographic or taxonomic restrictions. Our paper introduces the structure and features of ChiroVox and highlights the importance of further contributions to build an even larger and more comprehensive bat call library.

## Data integration pipeline

**Figure 1:**
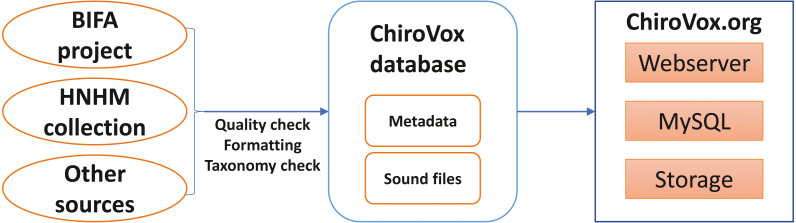
Schematic figure of the ChiroVox system.

Three main sources of sound recordings were available at the start of the project (Fig. 1). A considerable part of the information and sound files came from participants of a Data Mobilization Project awarded to the Southeast Asian Bat Conservation Research Unit (SEABCRU) by the Biodiversity Information Fund for Asia (BIFA) and from the collection of the Hungarian Natural History Museum (HNHM). Other sources were individual researchers who wanted to contribute to the project. The quality of the recordings and the metadata (including biodiversity data and recording circumstances) were reviewed by the site administrators and corrected if necessary. The taxonomy of species was checked by experts in the field based on the backbone suggested by Wilson & Mittermeier (2019), and current names were attached to the recordings. Checks were made to confirm that the species is known to occur in the area specified by the contributor. Following corrections, the recordings were integrated into the ChiroVox database. The sound files and the connected metadata were transferred into the storage and the MySQL database server of the ChiroVox website, respectively. The website was coded with the use of HTML, CSS, and PHP languages. Currently, new sound recordings can be submitted by contacting the site administrators.

## Metadata description

ChiroVox is an archive of bat echolocation and social call recordings, hence every uploaded file has a unique identifier (ChiroVoxUID), that can be cited in publications. This permits re-analysis of results in subsequent studies using the same recordings.

ChiroVox mostly provides reference calls that were recorded from bats identified with high certainty to family, genus and species. Taxonomic remarks can be added in cases where identifications are ambiguous. A taxonomic certainty score is required of all submissions. A score goes from 1–5, where 1 indicates greatest uncertainty about the taxonomic identity of the recorded bat. A 1 might be scored if the provider is not an expert, the bat was not identified in the hand, and comes from an area of high bat diversity and several similar species. A 5 would be selected if the identification was made by an expert and the animal was studied in the hand and/or there is voucher/genetic data, or the species’ calls are unique (like in many cases in temperate zones). The basis for each species identification (*e.g.*, voucher specimen, morphological measurement, photograph, genetic analysis) can also be added to the record.

The recordings can be connected to a voucher or to other databases like Global Biodiversity Information Facility (GBIF), National Center for Biotechnology Information (NCBI) GenBank, European Nucleotide Archive (ENA), Barcode of Life Data System (BOLD), *etc.* to facilitate interdisciplinary research. Majority of the metadata is in Darwin Core format to be compatible with several other important databases and services.

The most important biodiversity data can be also provided for a recording. This includes locality information (*e.g.*, country, territory, settlement, coordinates, *etc.*) as well as the date of recording (capture of bat). Other important factors that can also be given include the recording habitat, microhabitat structure, recording devices, recording method, and call type.

Recording bat sounds often requires significant efforts by researchers. Commercial use of recordings is generally prohibited in ChiroVox, although interested parties may contact the individual data providers to request permission. ChiroVox recordings can be accessed in two ways: (a) Open-access, whereby recordings and their metadata are freely available to the public for viewing and downloading under different Creative Common licenses; (b) Consensual-access, whereby associated metadata are available to the public and individual recordings can be requested from the contributor(s) on a case-by-case basis.

The complete list of the metadata variables and their explanations can be found in Table 2.

**Table 2:** Record variables used in the ChiroVox database. Variable groups are indicated by alternating colors.

Variable Name	Explanation
ChiroVoxUID	unique ID for recording (*e.g.,* A000001)
Family	bat family
Genus	bat genus
Species	bat species
Subspecies	bat subspecies
Taxonomic remarks	remarks on the taxonomy of the bat
Taxonomic certainty	certainty of the taxonomic identification (on a 1 to 5 scale, higher the better)
Identified by	person(s) who identified the bat
ID evidence	evidence(s) the identification is based on (*e.g.,* voucher, measurements, photo, *etc.*)
Gender	gender of the bat
Age	age of the bat
Individual #	individual number (any ID, *e.g.,* field number) of the bat
Voucher #	voucher number of the bat
Accession #	collection accession number of the bat
GBIF UID	GBIF unique identifier of the bat
Genetic #	accession number(s) belonging to the bat (in NCBI GenBank, ENA, BOLD, *etc.*)
Date	date of recording
Time	time of recording
Latitude	latitude in decimal degrees format
Longitude	longitude in decimal degrees format
Location accuracy	approx. accuracy of coordinates
Altitude	meters a.s.l. of the locality
Locality	the most exact place name where the bat was caught/recording took place
Settlement	settlement where the bat was caught/recording took place
Territory	province/county where the bat was caught/recording took place
Country	country where the bat was caught/recording took place
Habitat	habitat of the recording where applicable (*e.g.,* primary forest, river, tea plantation, *etc.*)
Microhabitat	structure of habitat, when applicable (*e.g.,* open, semi-cluttered, cluttered, *etc.*)
Method	recording method (*e.g.,* free-flying, hand held, hand release, enclosure, emergence, etc.)
Call type	type of the bat call (*e.g.,* search, feeding, social, distress, *etc.*)
Recording quality	quality of the recording (on a 1 to 5 scale, higher the better)
Device manufacturer	manufacturer of the recording device
Device model	model of the recording device
Sampling rate	sampling rate of the recording for real time and time expansion systems, in kHz
Recording type	type of recording (*e.g.,* real time, time expansion, *etc.*)
TE factor	time expansion factor
FD factor	frequency division factor
Access type	access type (*e.g.,* CC BY-NC 4.0, restricted, *etc.*)
Recordist name	name of the person(s) who recorded the call
Contact	name of the person(s) who must be contacted regarding the record
Contact e-mail	e-mail of the person(s) who must be contacted regarding the record
Reference	citation(s) of the publication(s) in which the species record or/and call description has been published
Remarks	additional information which does not fit in other categories

## Website capabilities

The website provides a summary of data holdings, including the total number of recordings, taxonomic and geographic coverage. A list of bat species and number of recordings for each taxon is also provided. The database can be browsed by species name, country, contributor or ChiroVoxUID. Every recording has a separate page where the metadata can be viewed and the sound file can be downloaded.

As we wish to encourage interest in the world of bat acoustics, the ChiroVox website provides links for further information on different equipment and software used to record and analyze bat sounds. Links to other bat call libraries can be found which may be helpful to find recordings of taxa not yet available on ChiroVox. Lists of the most important publications (*e.g.*, identification keys) and papers citing either ChiroVox or calls that are accessible through the website are available.

## Current holdings

At the time of submission, 3,902 bat calls are available through the website, representing 11 bat families, 46 genera, and 192 species (Table 3). To the best of our knowledge, more than 150 of these species are not represented in other online sources.

**Table 3:** Families and number of genera and species currently represented in ChiroVox.

Family	Genera	Species
Emballonuridae	3	6
Hipposideridae	4	28
Megadermatidae	2	2
Miniopteridae	1	6
Molossidae	3	3
Nycteridae	1	2
Pteropodidae	4	5
Rhinolophidae	1	36
Rhinopomatidae	1	1
Vespertilionidae	26	103
**Total**	**46**	**192**

**Figure 2:**
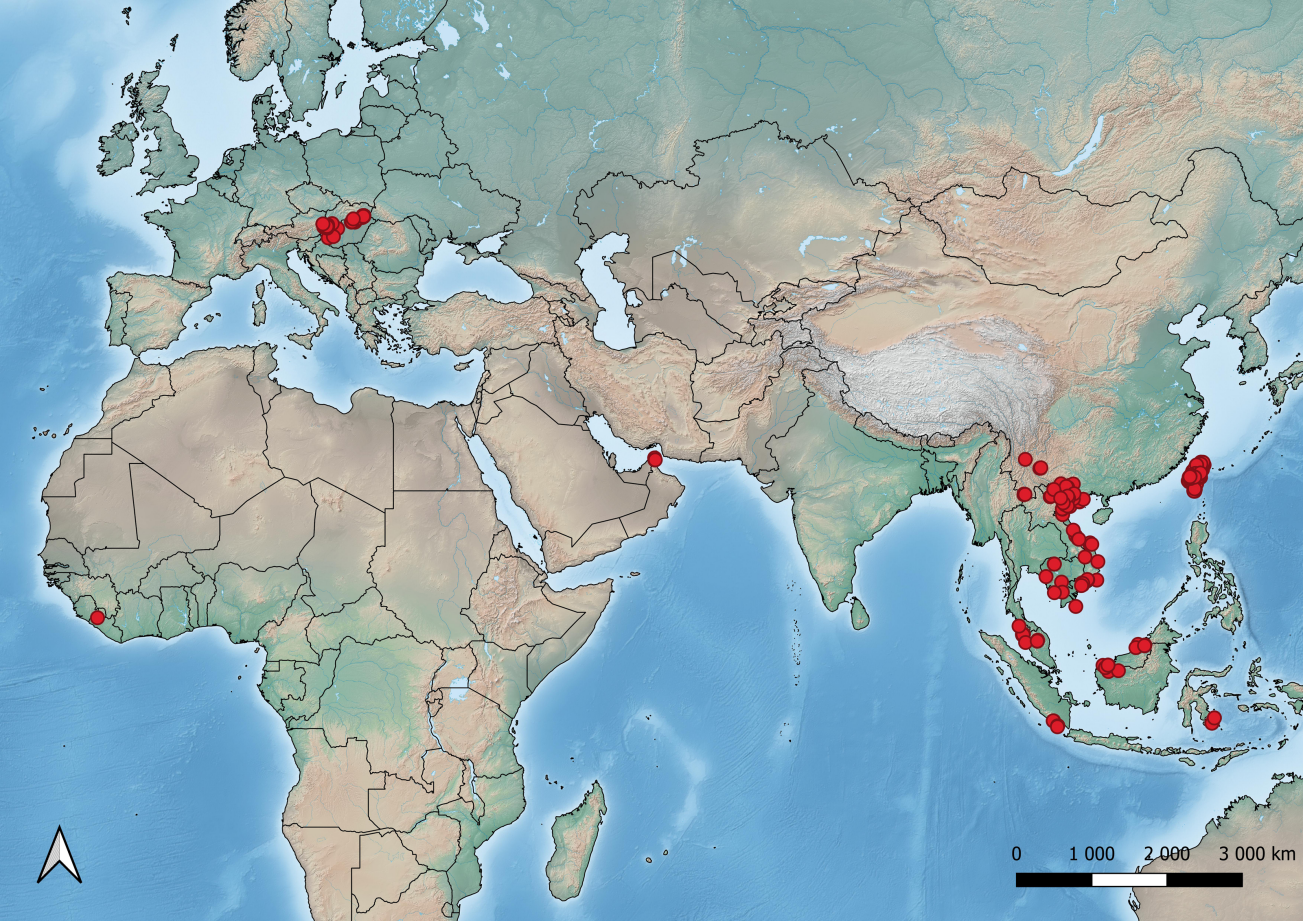
Geographic coverage of recordings available through ChiroVox. Red solid circles indicate locations of recordings currently available in the database.

Altogether more than 30 researchers contributed to the ChiroVox dataset which includes recordings from nine countries (Cambodia, China, Hungary, Indonesia, Liberia, Malaysia, Taiwan, United Arab Emirates and Vietnam) (Fig. 2).

## Discussion

To the best of our knowledge, ChiroVox is the largest library of bat recordings that are freely available through the internet. With almost 200 bat species represented in over 3,900 recordings, it is an important resource for bat acoustic studies.

Most of the recordings available through the website are from bats identified with high certainty, hence they can be used to determine the identity of anonymous bats in new recordings. This is especially important in regions where bat diversity is high and species identification often requires special taxonomic knowledge. We applied the most recent taxonomic backbone (Wilson & Mittermeier, 2019) to our database and our experts checked every submission to ensure its taxonomic integrity. Recognizing that Wilson & Mittermeier (2019) will be not updated, in the future we will use Simmons & Cirranello (2020) as a complementary taxonomic reference.

The species identification of free flying bats may largely depend on the circumstances of the reference recordings. At present, nearly 75% of the recordings in the ChiroVox library were made in closed spaces or from hand-held bats as specimens were often taken to identify them to species level. The majority of these recordings are of rhinolophids and hipposiderids (∼1,600 recordings) which can be used for identification of free flying bats because the echolocation call frequency (*i.e.,* frequency of the constant frequency component) of these species are similar in different environments. However, several hundred recordings are of species whose call characteristics show large context-dependent plasticity. For example, call frequency and structure are known to be affected by recording methods (Siemers, 2004) and the (micro-) habitat of the recording site (Kingston et al., 2003; Russ, 2012). Therefore, these reference recordings should be used for identification of free-flying bats with consideration of the recording conditions. For the future, as faunas become better-known, and vouchered genetic material becomes available, people should be encouraged to record more of their catch on release and use tissue samples to voucher identity. This will result in recordings that are more useful in the identification of free-flying bats.

The taxonomic coverage of the library at the time of launch includes nearly half of the ∼300 echolocating species that occur in Southeast Asia. Most of these recordings are from bat species that lacked a publicly available recording to date. These include several species recently described such as *Aselliscus dongbacanus*, *Glischropus aquilus*, *Hipposideros kunzi*, *Kerivoula dongduongana*, several *Murina* species, *Tylonycteris tonkinensis* and for example *Mirostrellus joffrei*, currently the sole representative of a new genus split from *Hypsugo* (Görföl et al., 2020). Several recordings are also available from bats that are yet to be formally described, such as *Phoniscus* sp., *Submyotodon* sp. *etc.* The library may facilitate the identification of less known species resulting in more occurrence records and more complete faunal inventories. It may also lead to the exploration of geographic variations (*e.g.*, Ith et al., 2015), help to solve complex taxonomic problems (*e.g.*, Tu et al., 2017) and provide insights into the role of echolocation in bat speciation (*e.g.*, Kingston & Rossiter, 2004).

The website has many recordings that were published in papers describing the echolocation call parameters of different species (Furey, Mackie & Racey, 2009; Phauk, Sarith & Furey, 2013; Huang et al., 2014; Huang et al., 2019; Shazali et al., 2016; Görföl et al., 2020; Kao et al., 2020; Donnelly et al., 2021; McArthur & Khan, 2021). Although the ChiroVoxUID was not available when these papers were published, it is now possible to cite calls with their UIDs in new publications, ensuring that studies based on call analyses will be replicable. Integration of other associated identifiers (*e.g.*, GBIF UID, voucher and sequence accession numbers) allows future publications to link the calls to published records, which are extremely important as the taxonomy of bats for some regions, *e.g.*, SE Asia, has changed rapidly in the past few decades.

The development of ChiroVox is continuous, hence more features will become available in the future. We would like to apply a user management system, whereby anybody can register and upload their recordings into the database. This will be a huge step forward, because the sharing of the recordings will be much easier and require less contributions from the maintainers of the website. The user management system will also allow easier sharing of “restricted” calls because a contributor can grant access to specific users to download specific recordings. The batch upload function for importing metadata will allow the sharing of larger datasets, whereas an option to batch download files will make it easier to download several recordings simultaneously. Long-term sustainability of ChiroVox is secured by the support of the Hungarian Natural History Museum.

The website is community based; hence every contribution is highly welcome as this is the only way it can grow. We are particularly interested in recordings from, and curators of other regions, especially from other least-known areas like Africa, and the tropics of America and South Asia.

## Conclusions

We built the ChiroVox website to facilitate the sharing of bat sound recordings from all over the world. More than 3,900 recordings of nearly 200 species are already deposited, making ChiroVox the largest open-access bat call library currently available. To the best of our knowledge, more than 150 of these species are not represented in other online sources. Each recording has a unique identifier which allows its citation in publications. Most of the recordings are from bats whose species identities are confirmed, so they can be used as a reference for determination of unknown recordings. We hope that with the help of the bat researcher community, the website will grow rapidly and will serve as a solid source for a wide variety of bat acoustic research and monitoring.

## Additional Information and Declarations

### Data Availability

The following information was supplied regarding data availability: The data is available at https://www.chirovox.org.
